# Effects of upper arch expansion using clear aligners on different stride and torque: a three-dimensional finite element analysis

**DOI:** 10.1186/s12903-023-03655-y

**Published:** 2023-11-20

**Authors:** Yanqi Zhang, Shuoyi Hui, Linyuan Gui, Fang Jin

**Affiliations:** State Key Laboratory of Oral & Maxillofacial Reconstruction and Regeneration & National Clinical Research Center for Oral Diseases & Shaanxi Clinical Research Center for Oral Diseases & Department of Orthodontics, School of Stomatology, Air Force Medical University, Xi’an, Shaanxi 710032 China

**Keywords:** Arch expansion, Clear aligner, Finite element, Torque, Stride length

## Abstract

**Background:**

During maxillary arch expansion with a clear aligner (CA), buccal tipping of the posterior teeth often occurs, resulting in an unsatisfactory arch expansion effect. The aim of this study was to analyze the appropriate maxillary arch expansion stride length and torque compensation angle for maxillary dentition to achieve an ideal moving state when a CA was used for upper arch expansion.

**Methods:**

This study established a three-dimensional (3D) finite element model including a CA, maxilla, periodontal ligament (PDL), and maxillary dentition. The stress distribution, stress situation, expansion efficiency, and movement trends of the maxillary dentition during upper arch expansion of different stride (0.1 mm, 0.2 mm, and 0.3 mm) and torque compensation (0°, 0.5°, 1°, and 1.5°) were measured.

**Results:**

Maxillary arch expansion lead to buccal tilt of the posterior teeth, lingual tilt of the anterior teeth, and extrusion of the incisors. As the angle of compensation increased, the degree of buccal tilt on the posterior teeth decreased, with this reducing the efficiency of upper arch expansion. When the stride length was 0.1 mm, the torque compensation was 1.2°, and when stride length was 0.2 mm and the torque compensation was approximately 2°, there was a tendency for the posterior teeth to move bodily. However, when the stride length was 0.3 mm, the increase in torque compensation could not significantly improve the buccal tilt phenomenon. In addition, the equivalent von-Mises stress values of the maxillary root, PDL, and alveolar bone were in the same order of magnitude.

**Conclusions:**

This study indicated that the posterior teeth cause a degree of buccal tilt when maxillary arch expansion is ensured. The specific torque compensation angle should be determined based on the patient’s situation and the desired effect.

**Supplementary Information:**

The online version contains supplementary material available at 10.1186/s12903-023-03655-y.

## Background

In recent years, clear aligner therapy (CAT) has been used widely in orthodontic treatment due to its aesthetic appeal and comfort. However, clear clinical recommendations and standards for CAT based on scientific evidence have not yet been established [1]. For example, designing the target position in CAT does not always result in the expected tooth movement [2], with unpredictability being common [3].

Expanding the upper arch with CAT is a viable option [4] for many patients with a limited transverse maxillary deficiency or mild to moderate dental crowding as it provides space to solve crowding, and also improves the bite by matching the upper and lower dentition. Although CAT is widely popular, it still has limitations in the field of arch expansion. Some studies have pointed out that fixed appliances improve malocclusion more effectively than that achieved by Invisalign [5, 6]. In addition, statistical analyses have shown that the efficiency of maxillary arch expansion correlates negatively with the preset amount of expansion movement [7, 8]. Most importantly, maxillary arch expansion is achieved mainly through the tipping movement of posterior teeth, with the tilt angle increasing as the arch expands [8, 9].

However, posterior buccal tilt can cause posterior palatal tip drooping, leading to occlusal disorders and vertical problems, and potentially periodontal problems, such as alveolar bone resorption. Although the efficiency of arch expansion and teeth movement patterns by CAT are not satisfactory, only a small number of studies have investigated the technique in detail. It is therefore crucial to gain a better understanding of the basic biomechanical mechanism of maxillary arch expansion caused by CAT in order to effectively control the movement of teeth during clinical treatment.

The 3D finite element method (FEM) is a computer technique that simulates the stress distribution of the PDL and alveolar bone after application of loads that simulate teeth displacement [10]. This technique has been used extensively in the field of orthodontics to help gain a better understanding of the biomechanics involved in orthodontic movement and also to provide guidance for clinical operations.

The current study used FEM to analyze the biomechanics of maxillary arch expansion and to investigate the appropriate stride length and torque compensation angle. This information was then used to guide the clinical use of clear aligners in upper arch expansion.

## Methods

### Construction of the 3D finite element model

**Fig. 1:**
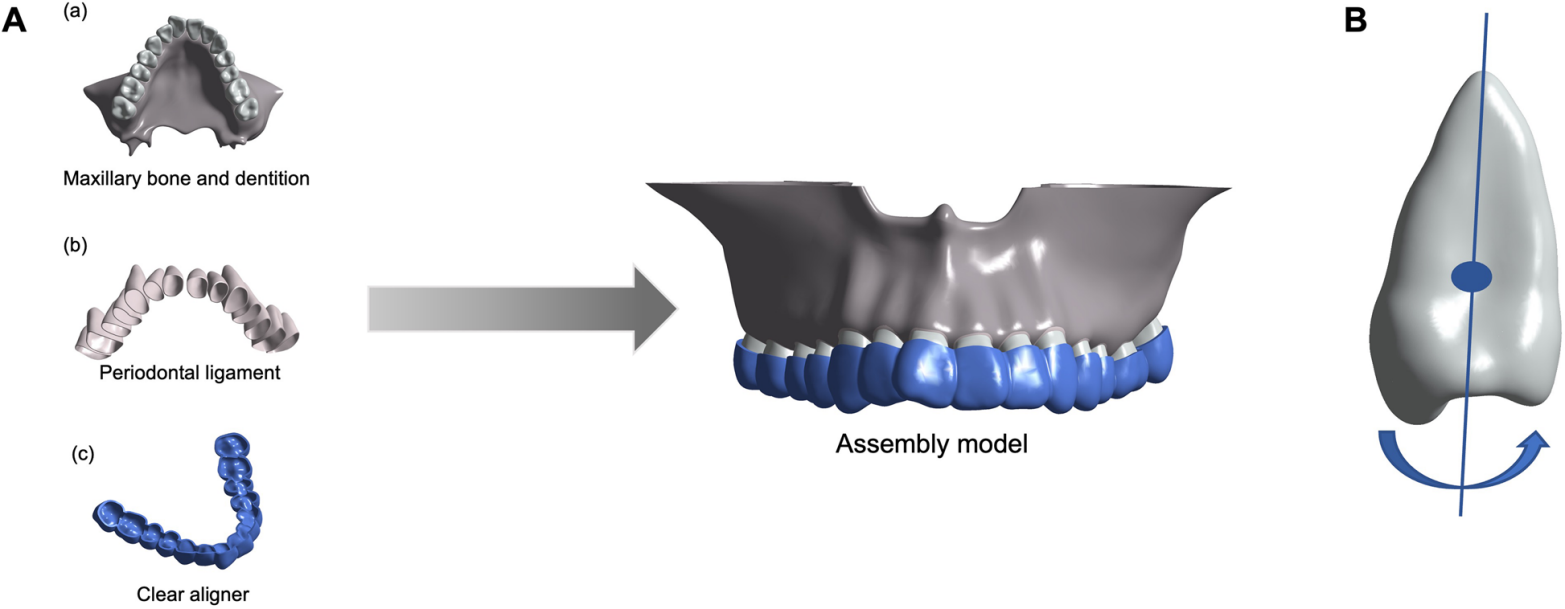
Computer-aided designed models. **A** The 3D finite element model including (a) Maxillary bone and dentition (gray), (b) PDL (dusty pink), and (c) CA (blue). **B** Torque compensation was centered near the tooth neck, with the tooth crown rotated towards the palatal side

Cone-beam computed tomography (CBCT) data (GE Healthcare, USA) were obtained from a patient with a narrow dental arch who had mildly congested dentition with generally normal axial inclination of posterior teeth and mild torsion of individual maxillary teeth (Fig. 1A). The CBCT scan was performed with full FoV, a centered rotation of 360°. The voltage and current were 100 kV, 4 mA, and the exposure timing was 15 s. The thickness of each CBCT section was set to 0.15 mm. The pixel size of CBCT is 0.25 mm.

As shown in Fig. 1A, Mimics 21.0 software (Materialise Software, Leuven, Belgium) was used to extract and build the maxilla and upper dentition model of the patient. Geomagic Wrap 2021 (3D Systems, North Carolina, USA) was then used to optimize the original 3D model and create the surface model structure. With the help of 3D mechanical drawing software NX 1911 (Siemens, Germany), the maxillary root was extended outward by 0.25 mm to build the PDL model. Cortical bone and cancellous bone models were established by moving the maxilla inward by 1.3 mm, while the maxillary crown was extended outward by 0.75 mm to simulate the thickness of the CA. All the components are then imported into the ANSYS Workbench 2021 (Ansys, Pennsylvania, USA) to generate a 3D finite element model, which is a 3D 10-node tetrahedral structural solid of the case for subsequent analysis.

### Properties and boundary conditions of materials

The properties of the material were adopted from previously published research [11–14] (Table 1). The superior region of maxilla is set as the boundary, and the movement of the maxilla bone is restricted for all degrees of freedom. The root was bound to the periodontal membrane, while the periodontal membrane was bound to the alveolar bone without sliding. The structure was assumed to be isotropic and homogeneous. The contact relationship between the CA and the tooth was assumed to be nonlinear, with a friction coefficient of 0.2 [15]. All other contact relationships were assumed to be linear, with no separation between the crowns and frictionless sliding. The PDL was set-up as a viscoelastic, nonlinear structure based on previous studies [16, 17]. The current study therefore belongs to the category of nonlinear research.

**Table 1:** Material properties

Component	Young’s modulus (MPa)	Poisson’s ratio
Teeth	1.96 × 10^4^	0.30
PDL	6.9 × 10^−1^	0.45
Cortical bone	1.37 × 10^4^	0.30
Cancellous bone	1.37 × 10^3^	0.30
Clear Aligner	5.28 × 10^2^	0.36

### Experiment design

We applied a stride length of 0.1 mm (A), 0.2 mm (B), and 0.3 mm (C) in our study to simultaneously expand the bilateral posterior teeth in the maxilla. To compensate for the torque, we set torque compensation values of 0°, 0.5°, 1.0°, and 1.5° for groups A, B, and C, respectively (refer to Table 2 for the grouping details). Torque compensation was centered near the tooth neck (the junction of crown enamel and root cementum), with the crown rotated towards the palatal side along the long axis of tooth (Fig. 1B). The CA model was then expanded outwards by 0.75 mm according to the subsequent movement of the dentition. Finally, we overlaid the CA model on the initial dentition model to generate orthodontic forces.

**Table 2:** Grouping situation

Group	Stride length (mm)	Torque compensation (°)
A1	0.1	0
A2		0.5
A3		1.0
A4		1.5
B1	0.2	0
B2		0.5
B3		1.0
B4		1.5
C1	0.3	0
C2		0.5
C3		1.0
C4		1.5

### Outcomes

The finite element model mesh was divided, with the number of nodes and elements in all the groups shown in Table 3. We established two global coordinate systems in this study. In the coordinate system of the entire maxillary dentition, the X-axis represented the coronal plane (positive on the left side and negative on the right side), the Y-axis represented the sagittal plane (positive on the posterior and negative on the anterior), and the Z-axis represented the vertical plane (positive on the superior and negative on the inferior). In the coordinate system of a single dentition, the X-axis was the mesiodistal axis (the mesial direction of dentition in region 1 was positive and the distal direction was negative; Zone 2 dentition is opposite). The Y-axis is represented the labiolingual axis (positive on the palatal side and negative on the buccal side), while the Z-axis represented the vertical axis (intrusion was positive and extrusion was negative). The displacement of each tooth in three directions was then recorded, with the angle of inclination measured by the cross angle of the long axis of the tooth at the initial and final positions. Von-Mises stress of the alveolar bone, root, and PDL were analyzed by FEM.

**Table 3:** Nodes and elements

	A1	A2	A3	A4	B1	B2	B3	B4	C1	C2	C3	C4
Nodes	580,299	572,565	573,020	573,716	573,026	573,067	573,107	574,035	573,695	573,730	574,065	573,300
Elements	321,840	317,486	317,760	318,345	317,759	317,804	317,830	318,540	318,307	318,372	318,536	318,098

## Results

**Fig. 2:**
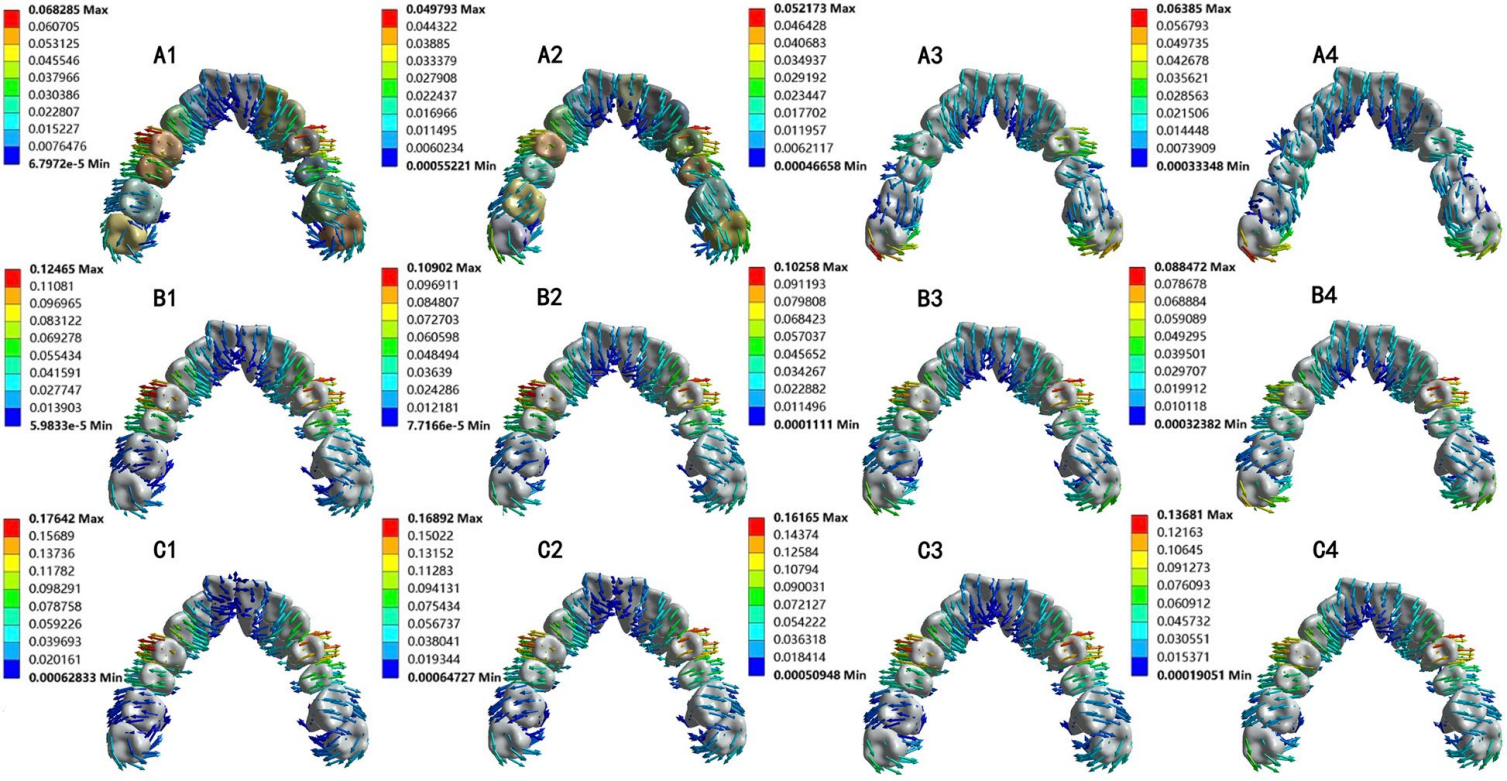
Displacement trend of maxillary total dentition after maxillary arch expansion in 12 groups (unit: mm)

The identical bilateral teeth exhibited a symmetric displacement pattern (Fig. 2). Therefore, in the subsequent analysis, the right teeth were selected to represent the movement trends and stress distribution of the identical bilateral teeth. In addition, as shown in Fig. 2, the second molars of each group rarely produced the maxillary arch expansion effect, so only the premolars and the first molars were analyzed in the subsequent analysis on the effect of upper arch expansion.

### The displacement and efficiency of the maxillary dentition in arch expansion without torque compensation

**Fig. 3:**
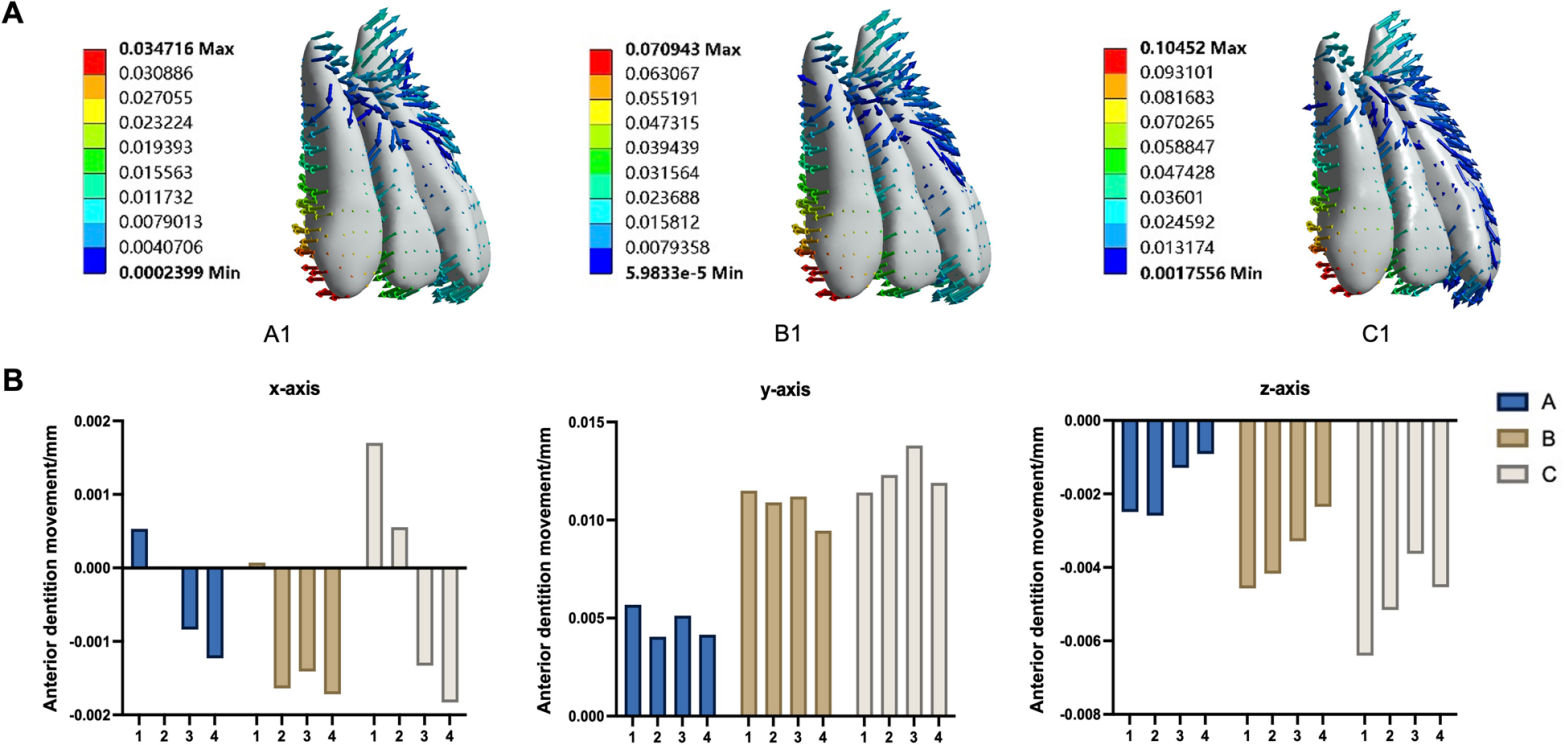
The displacement of anterior teeth (unit: mm). **A** The A1, B1, and C1 groups’ displacement trend diagram of the upper anterior teeth after arch expansion. **B** The bar graph analysis of the upper anterior teeth after arch expansion with different stride length and torque compensation

**Fig. 4:**
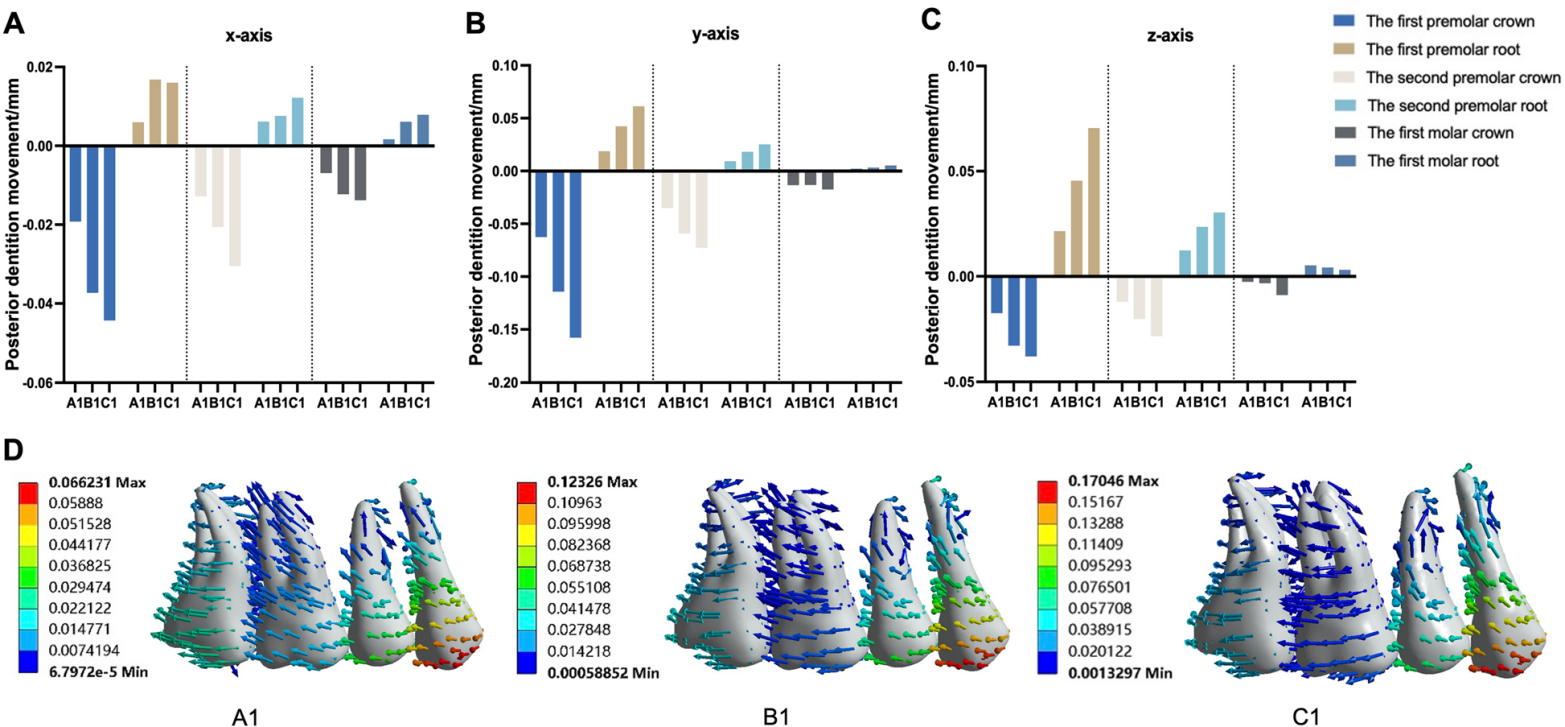
Displacement tendencies of posterior teeth when maxillary arch expansion without torque compensation (unit: mm). **A-C** The bar graph analysis of the posterior teeth after maxillary arch expansion. **D** The A1, B1, and C1 groups’ displacement trend diagram of the anterior teeth after maxillary arch expansion

**Fig. 5:**
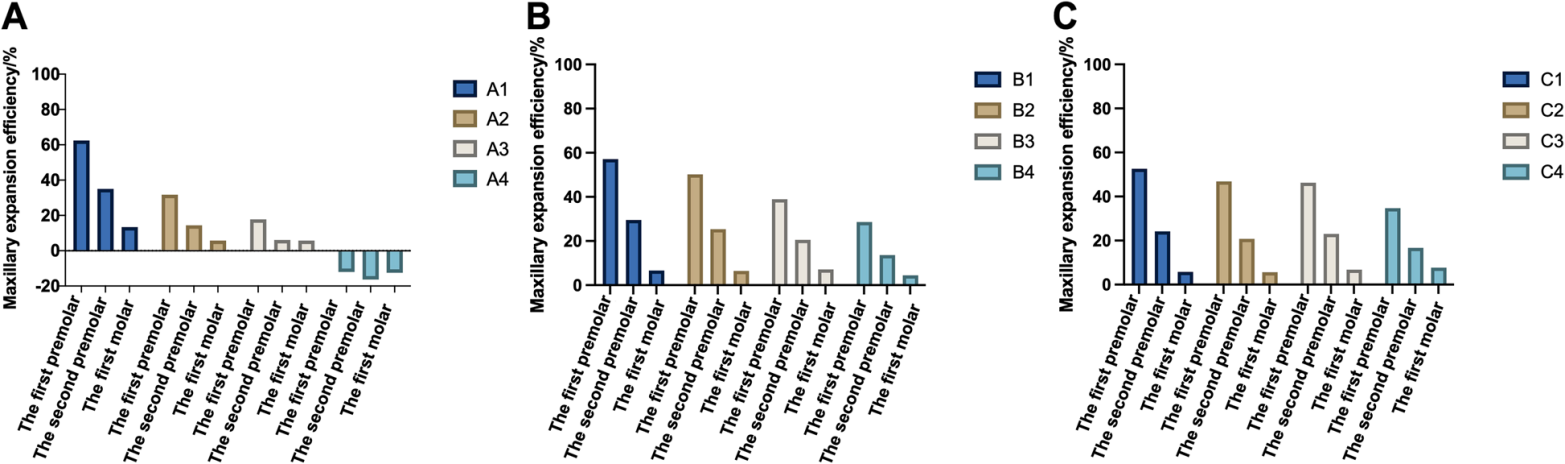
Posterior teeth displacement efficiency of maxillary arch expansion. **A** The expansion stride length is 0.1 mm. **B** The expansion stride length is 0.2 mm. **C** The expansion stride length is 0.3 mm

According to the coordinate system mentioned above, when the maxillary arch was expanded, the anterior teeth tilted lingually, while the incisor teeth were extruded (Fig. 3). Upper arch expansion was achieved by buccal tipping of the posterior teeth with a small tendency for distal tipping (Fig. 4D). With an increase in the stride length of the upper arch expansion, the posterior teeth tilted more significantly to the buccal and distal sides (Fig. 4A-C). As shown in Fig. 5, the efficiency of the maxillary arch expansion decreased from the first premolar to the first molar, with this efficiency decreasing with an increase in stride length.

### Changes in the posterior teeth after designing the torque compensation angle

**Fig. 6:**
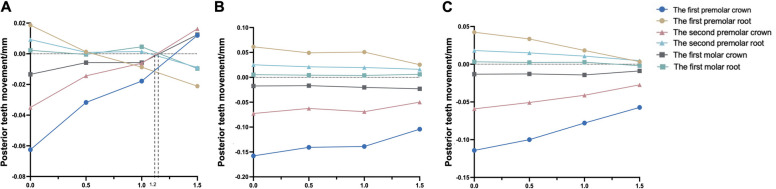
Prediction when bodily movement for the posterior teeth with torque compensation at different stride lengths (unit: mm). **A** The expansion stride length is 0.1 mm. **B** The expansion stride length is 0.2 mm. **C** The expansion stride length is 0.3 mm

As depicted in Table 4, the degree of buccal inclination of the posterior teeth decreased from the first premolar to the second molar. The influence of different torque compensation on the degree of posterior tooth inclination also decreased from the first premolar to the second molar. After torque compensation was added, the degree of buccal tilt in the posterior teeth was reduced and even palatal tilt appeared, such as in the first molar of groups A3 and A4. As shown in Fig. 6, with an increase in torque angle, the buccal movement of the posterior tooth crown gradually decreases, and the palatal movement of the root also gradually decreases or even moves to the buccal side.

**Table 4:** The displacement, tilt angle, and highest equivalent von-Mises

	The displacement of anterior teeth X y z (unit: mm)	The tilt angle of the first premolar, second premolar, first molar, and second molar (unit: °)	The highest equivalent von-Mises value of the posterior teeth root, PDL, and alveolar bone (unit: MPa)
A1	(0.0005, 0.0057, − 0.0025)	−1.29, − 0.60, − 0.27, 0.024	0.060,0.011, 0.0035
A2	(0.000019, 0.0041, − 0.0026)	− 0.64, − 0.23, − 0.078, 0.20	0.051, 0.0068, 0.0026
A3	(− 0.00084, 0.0051, − 0.0013)	−0.32, 0.0058, 0.078, 0.53	0.075, 0.0084, 0.040
A4	(−0.0012, 0.0042, − 0.00091)	0.097, 0.27, 0.17, 0.65	0.11, 0.011, 0.049
B1	(0.000071, 0.011, − 0.0046)	− 2.4, − 1.0, − 0.20, 0.29	0.12, 0.027, 0.22
B2	(− 0.0016, 0.011, − 0.0042)	− 2.1, − 0.88, − 0.18, 0.50	0.092, 0.023, 0.17
B3	(−0.0014, 0.011, − 0.0033)	− 1.6, − 0.69, − 0.21, 0.67	0.080, 0.022, 0.13
B4	(−0.0017, 0.0095, − 0.0024)	−1.2, − 0.43, − 0.14, 0.81	0.12, 0.018, 0.15
C1	(0.0017, 0.011, − 0.0064)	−3.3, − 1.2, − 0.26, 0.33	0.23, 0.049, 0.55
C2	(0.00055, 0.012, − 0.0052)	−2.9, − 1.1, − 0.24, 0.51	0.20,0.042, 0.47
C3	(−0.0013, 0.014, − 0.0036)	− 2.9, − 1.2, − 0.29, 0.90	0.22, 0.041, 0.37
C4	(−0.0018, 0.012, − 0.0045)	−2.2, − 0.83, − 0.37, 0.82	0.16, 0.033, 0.36

In groups A and B, the arch expansion efficiency decreased with an increase in torque compensation angle. When the stride length was 0.1 mm, the torque compensation was 1.5°, which caused the efficiency of the posterior teeth to become negative and not achieve the purpose of expanding the arch. However, in group C, the arch expansion efficiency tended towards a constant value and was rarely affected by the torque compensation angle (Fig. 5).

### The bodily movement of posterior teeth under different treatments during maxillary arch expansion

As displayed in Fig. 6, we concluded that when the stride length was 0.1 mm, there was bodily movement of the first molar when the torque compensation was 1.3° and bodily movement of the premolars when the torque compensation was 1.2°. When the stride length was 0.2 mm, we also inferred that a torque compensation of 1.6° on the first molar achieved bodily movement, with movement occurring for the first premolar when the torque compensation was 2.35° and for the second premolar when the torque compensation was 2.15°. However, when the step length reached 0.3 mm, the torque compensation had no obvious effect on the movement of the posterior teeth, and the tilt movement achieved an arch expansion effect.

### The equivalent von-Mises stress values of the root, PDL and alveolar bone

**Fig. 7:**
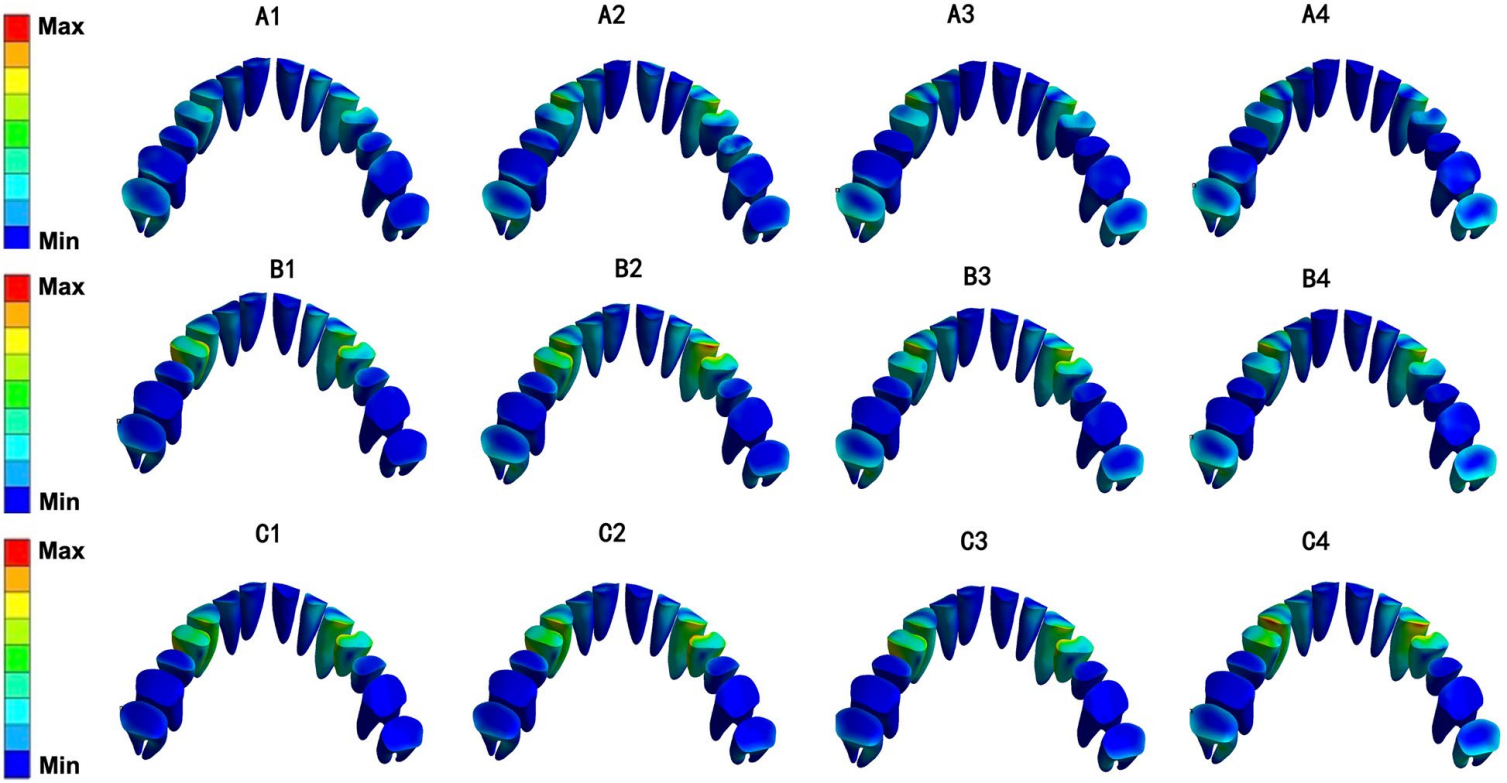
Equivalent von-Mises stress of root of maxillary teeth (unit: MPa)

**Fig. 8:**
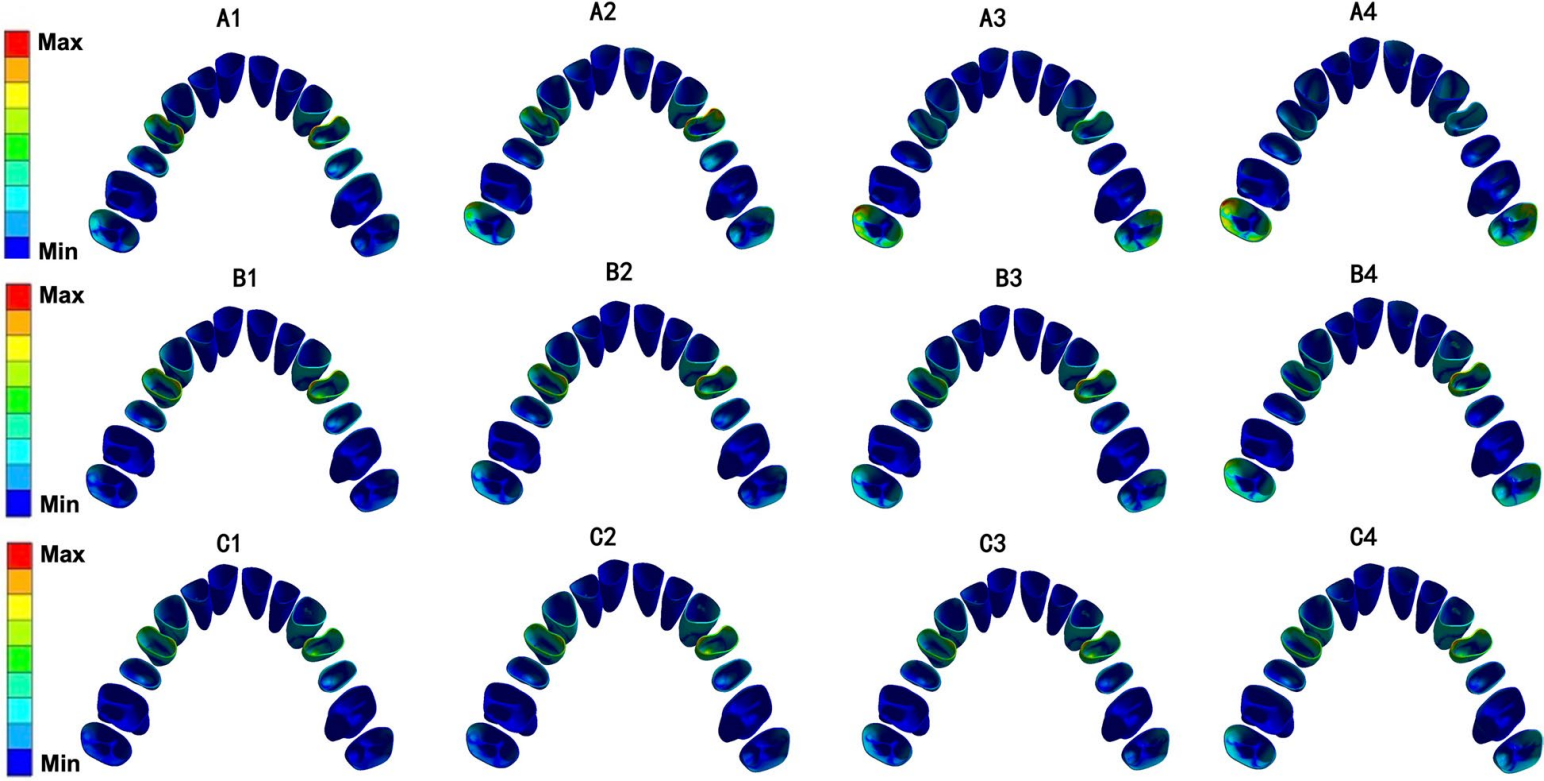
Equivalent von-Mises stress of PDL of maxillary teeth (unit: MPa)

**Fig. 9:**
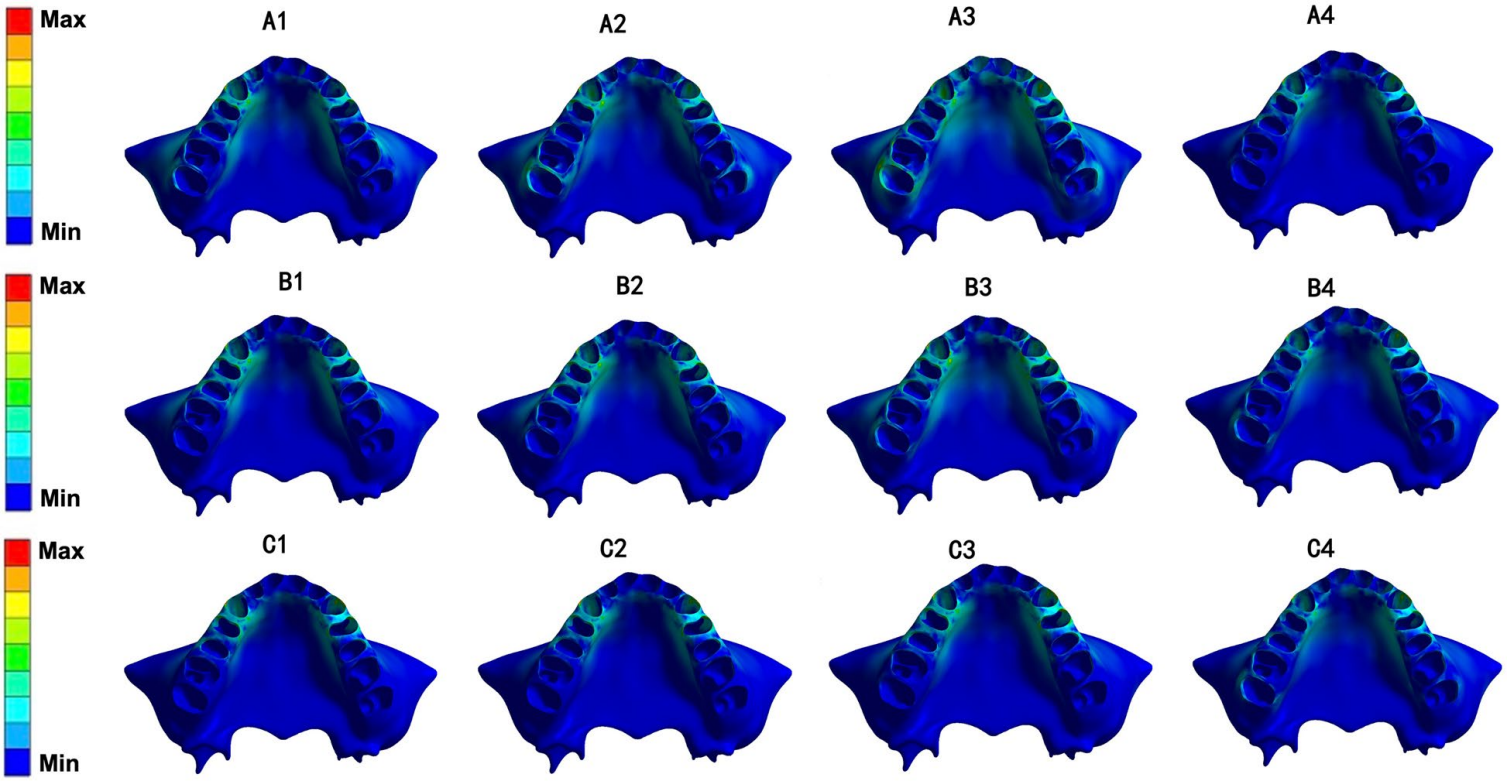
Equivalent von-Mises stress of alveolar bone (unit: MPa)

**Fig. 10:**
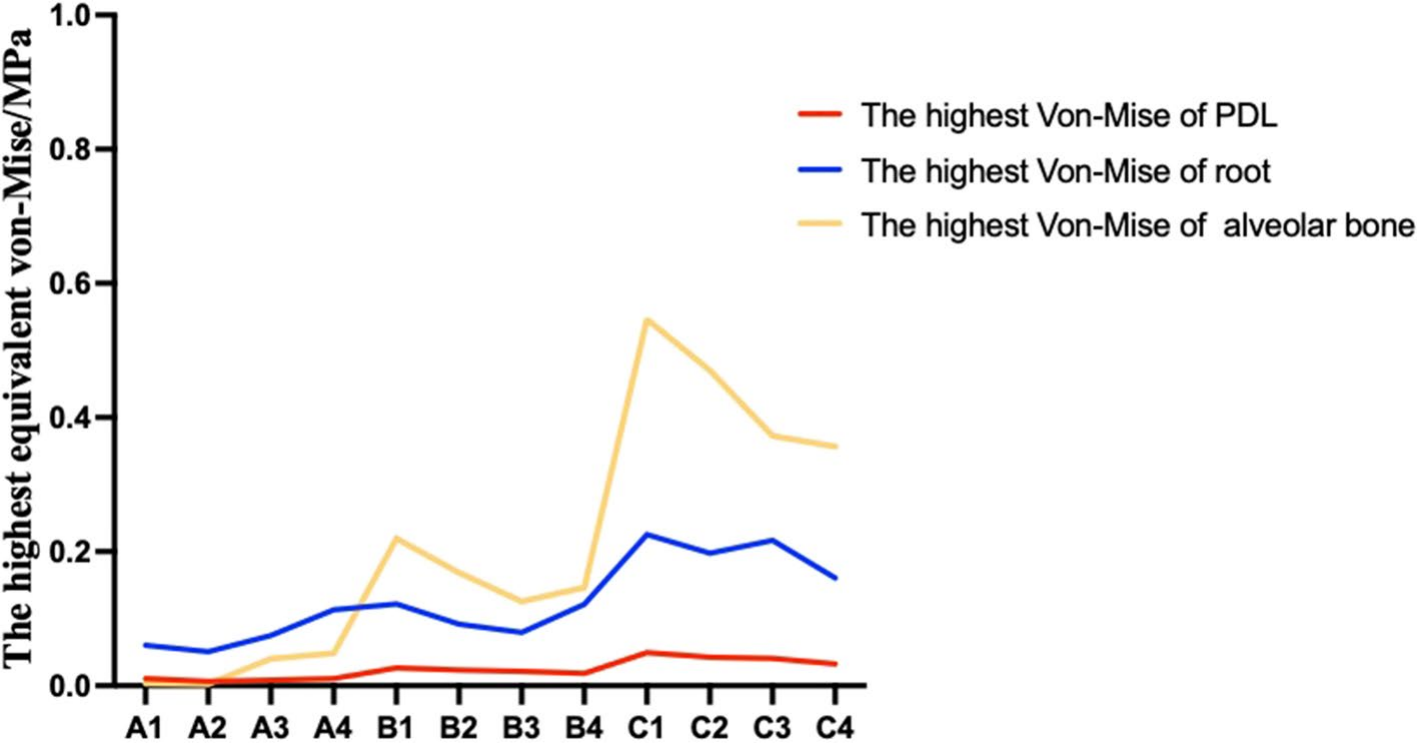
The highest equivalent von-Mises value of the posterior teeth root, PDL, and alveolar bone with different stride length and torque compensation when maxillary arch expansion (unit: MPa)

In all 12 models, the equivalent von-Mises stress value of the root was concentrated on the canine and first premolar and was more obvious in the cervical region (Fig. 7). The von-Mises stress value of the PDL was distributed mainly on the cervical region of the first premolar, while stress on the second molar showed an upward trend as the angle of the torque compensation increased (Fig. 8). The von-Mises stress value for the alveolar bone was concentrated mainly in the palatal region of the canine and first premolars and in the alveolar fossa of the canine (Fig. 9). The von-Mises stress value rose with an increase in stride length, but were in the same order of magnitude (Fig. 10).

## Discussion

CAT is used widely used in the treatment of malocclusion. Therefore, the biomechanical analysis of teeth movement is crucial in CAT. In recent years, with the rapid development of computer technology, FEM has been used as an effective tool in orthodontic biomechanics [10, 12, 18]. This study created a 3D finite element model of a patient with maxillary arch stenosis to investigate the appropriate stride length and torque compensation for maxillary arch expansion.

Clinical studies have demonstrated that the buccal tilt and efficiency of the posterior teeth decreases from the first premolar to the first molar when the upper arch expansion was created using CAT [7–9]. These findings are consistent with those of our study, which showed CA produced buccal and distal tipping of the posterior teeth, lingual tipping of the anterior teeth, and extrusion of the incisors with maxillary arch expansion without torque compensation. However, these trends in teeth movement may easily cause an occlusal disorder and mandibular clockwise rotation, which are unfavorable to the profile of patients with a mandibular retraction. If the tilt degree reaches a certain degree, it will also cause alveolar bone absorption. In order to reduce the above adverse effects, many clinicians increase the torque compensation during maxillary arch expansion. However, there is no scientific evidence on the specific degree of increase, with this chosen based on the experience of the physician.

Nowadays, many research focus on the transverse arch dimension, which is enough to prove the importance of transverse dimension of arch in orthodontics. Some scholars report that Mixed Palatal Expansion (MPE) can improve the transverse dimension of upper and lower arch [19]. Although there is evidence that CA is inferior to fixed orthodontics in orthodontic treatment for controlling teeth torque and increasing transverse width [5], the advantage of CAT is that it sets specific stride lengths and torque compensation. Therefore, we set-up different upper arch expansion stride lengths and torque compensation to help orthodontists understand the biomechanical mechanism of maxillary arch expansion.

The tilt angle was used in the current study to directly reflect the buccal tilt of the posterior teeth after increasing torque compensation. As displayed in Table 4, when no torque compensation was added, the maximum tilt angle of group A did not exceed 1.5°. With an increase of the stride length, the degree of buccal tilt of the posterior teeth increased successively, with a maximum of more than 3° when the stride length reached 0.3 mm. Therefore, we consider that it is reasonable to reduce the stride length to 0.1 mm in upper arch expansion when the posterior dental axis of the patient is normal or the periodontal condition is not ideal. Although the degree of buccal tilt of the posterior teeth decreased with an increase in torque compensation, when the stride length was 0.1 mm and the torque compensation was 1.5°, the posterior teeth crown moved to the palatal side. This indicated that an increase in torque angle will also reduce the efficiency of the posterior teeth in maxillary arch expansion.

Therefore, in addition to considering the degree of tilt, the effect of the expansion of the posterior teeth was also used as a reference factor. However, Fig. 6 shows that when the stride length was 0.1 mm and the torque compensation was 1.2°, the posterior teeth tended to demonstrate bodily movement to achieve the upper arch expansion effect. In addition, as shown in Fig. 5, the efficiency of upper arch expansion in this group was too low, with the highest being less than 18%, with the second premolar and molar teeth being lower. According to our speculation when the stride length was 0.2 mm, if we blindly pursued bodily movement, the efficiency of the maxillary arch expansion was greatly reduced. When the stride length was 0.3 mm, the degree tilt of the posterior teeth was severe and an increase in torque compensation did not improve it significantly. In addition, study have reported a risk of root resorption when hydrostatic pressure of PDL exceeds typical human capillary blood pressure. Root resorption may occur if the hydrostatic pressure rises above 0.0047 MPa, but other factors, such as the position of the pressure on the root, may also affect the severity of root resorption [20]. However, the authors emphasize that the tooth movement rate was low with a hydrostatic stress of 0.0047 MPa [21]. Although the highest Von-Mise in this study is different from the hydrostatic pressure mentioned above, based on the study on Von-Mise of tooth root resorption [22] and the stride length used in this study, we consider that the highest equivalent von-Mises stress values of the root, PDL, and alveolar bone were still within the normal physiological range. According to Fig. 10, the highest Von-Mise of PDL, root and alveolar bone increased significantly when the stride length reached 0.3 mm. Thus, we decided not to expand the upper arch using a 0.3 mm stride length. In conclusion, according to our comprehensive analysis of upper arch expansion efficiency and degree of tilt, we suggest that the step length should be 0.1 mm and the torque compensation 0.5°, or when maxillary arch expansion is required, the stride length should be 0.2 mm and the torque compensation 1.5°.

However, in clinical applications, different torque compensation should be set according to the specific situation of the patient. For patients who require a posterior buccal tilt, the effect of maxillary arch expansion is to correct the axial of the patient’s posterior teeth. For patients with severe buccal tipping, micro-implant assisted maxillary arch expansion [23] can be considered to avoid periodontal problems such as alveolar bone resorption. Some scholars have reported the difference between bone-borne Haas-inspired miniscrew-assisted maxillary expander (BB HIMAME) and bone-tooth-borne miniscrew-assisted rapid palatal expander (BTB MARPE) of mini-screw assisted maxillary expanders. The high stress around the fronto-maxillary suture of BB HIMAME, but the BTB MARPE will produce tipping movement of the anchor tooth [24]. This also reminds us that it is necessary to consider comprehensively when choosing micro-implant assisted maxillary arch expansion.

In addition to controlling the maxillary arch expansion stride length and torque compensation, we may also improve movement of teeth of the upper arch expansion in other ways. For example, some scholars have proposed that alternating movement can achieve higher movement efficiency than whole movement [25]. The thickness of the aligner also has a certain effect on teeth movement. Increasing the thickness of the appliance has been reported to result in more ideal movement of the target teeth [26]. In addition, academic and clinical observations have shown that although the use of attachments in upper arch expansion results in no significant difference in torque control of the posterior teeth, the use of attachments can increase aligner retention. Moreover, buccal attachment is routinely added in maxillary arch expansion by CAT. Studies have shown that simultaneous use of buccal and palatal attachments can control teeth movement patterns in molar intrusion [27]. This is also a concept that can be adopted in the future for maxillary arch expansion.

The current study also demonstrated that the anterior teeth will lingual tilt and the incisor will extrude during upper arch expansion. Interestingly, we showed that these movement trends were not affected by torque compensation, and only increased significantly when the expansion stride length was 0.2 mm (Fig. 3B). This suggested that corresponding treatment measures should be considered for the anterior teeth in CAT maxillary arch expansion, such as reducing the stride length and setting the torque compensation of the anterior teeth in order to avoid unpredictable tooth movement. Attention should also be paid to this issue in the future when using CAT to achieve maxillary arch expansion.

In summary, with a better understanding of the biomechanics of maxillary arch expansion by CAT we expect to achieve ideal movement of teeth in the future as CA materials advance. This improvement will make CA a convenient tool for orthodontists.

## Limitations

FEM is a static analysis which only reveals the displacement trends of teeth and stress distribution in the periodontal region. Teeth shape, cutting of the appliance edge, parameter setting, and contact mode of each part may affect the result of analyses. Therefore, follow-up research should also optimize the settings of various parts of the FEM, so that it is used better in the field of orthodontics. In addition, the conclusions of finite element studies should be used carefully in combination with a doctor’s own experience in clinical applications. Animal experiments and clinical prospective studies should be carried out to obtain more reliable evidence.

## Conclusion

This study discusses the effects of different stride lengths and torque compensation angles on maxillary arch expansion. Taking the limitations of the study into account we have made the following conclusions.

1. Maxillary arch expansion can cause lingual tipping of the anterior teeth, extrusion of the incisors, and the buccal and distal tipping of the posterior teeth. The above movements are enhanced by an increase in stride length.
2. Adding torque compensation was effective for controlling buccal tipping of the posterior teeth, but also led to a reduction in the efficiency of maxillary arch expansion.
3. Appropriate torque compensation such as 0.5° at 0.1 mm and 1.5° at 0.2 mm should be designed to ensure that the posterior teeth tend to bodily move and also to ensure a certain arch expansion efficiency.
4. With maxillary arch expansion, the anterior teeth should be controlled at the same time to avoid inappropriate movement.

### Supplementary Information

**Additional file 1.**

## Data Availability

The datasets used and analyzed during the current study are available from the corresponding author on reasonable request.

## Abbreviations

CAT: Clear aligner therapy
CA: Clear aligner
3D: Three-dimensional
FEA: Finite element analysis
PDL: Periodontal ligaments
CBCT: Cone beam computer tomography
MPE: Mixed palatal expansion
BB HIMAME: Bone-borne Haas-inspired miniscrew-assisted maxillary expander
BTB MARPE: Bone-tooth-borne miniscrew-assisted rapid palatal expander
